# Effects of Single Nucleotide Polymorphisms and Mediterranean Diet in Overweight or Obese Postmenopausal Women With Breast Cancer Receiving Adjuvant Hormone Therapy: A Pilot Randomized Controlled Trial

**DOI:** 10.3389/fnut.2022.882717

**Published:** 2022-07-01

**Authors:** A-Ra Cho, Kyung-Won Hong, Yu-Jin Kwon, Ja-Eun Choi, Hye-Sun Lee, Hyung-Mi Kim, Soong June Bae, Sung Gwe Ahn, Joon Jeong, Ji-Won Lee

**Affiliations:** ^1^Chaum Life Center, CHA University, Seoul, South Korea; ^2^Theragen Etex Bio Institute Co., Ltd., Suwon, South Korea; ^3^Department of Family Medicine, Yongin Severance Hospital, Yonsei University College of Medicine, Yongin, South Korea; ^4^Biostatistics Collaboration Unit, Department of Research Affairs, Yonsei University College of Medicine, Seoul, South Korea; ^5^Department of Food and Nutrition, Dongduk Women’s University, Seoul, South Korea; ^6^Department of Surgery, Gangnam Severance Hospital, Yonsei University College of Medicine, Seoul, South Korea; ^7^Department of Family Medicine, Severance Hospital, Yonsei University College of Medicine, Seoul, South Korea

**Keywords:** Mediterranean diet, *FTO* gene, *MC4R* gene, nutrigenetics, breast cancer

## Abstract

**Background and Aims:**

Weight management is recommended in overweight or obese breast cancer patients, as they have an increased risk of cancer recurrence and poor prognosis. Furthermore, identifying the relationships between genetic factors and nutrition could help suggest possible individualized nutritional solutions in weight management. The objective of this pilot randomized controlled trial was to investigate the influence of two obesity-associated single nucleotide polymorphisms and the Mediterranean diet intervention on weight loss and modification of nutrient intake and metabolic parameters in overweight or obese, postmenopausal, breast cancer patients receiving adjuvant hormone therapy.

**Methods:**

Seventy-eight breast cancer patients were randomly assigned to the Mediterranean diet (MeDiet) group or control group, and seventy-one were finally analyzed. Body composition, nutrient intake, and metabolic parameters were assessed at baseline and after the 8-week intervention. Fat mass and obesity-associated (*FTO*) rs7185735 and melanocortin-4 receptor (*MC4R*) rs476828 variants were genotyped.

**Results:**

We found that both variants did not influence weight loss or improvement of metabolic parameters within the Mediterranean diet intervention. Intake of saturated fatty acid (SFA) and trans fat was significantly increased in C carriers compared with the TT genotype of *MC4R* rs476828 only in the control group (*p* = 0.002 for SFA; *p* = 0.016 for trans fat), whereas no significant difference was observed between genotypes in the MeDiet group. There were statistically significant interactions between *MC4R* rs476828 and dietary intervention for changes in SFA intake (*p* = 0.009) and trans fat intake (*p* = 0.049).

**Conclusion:**

Our data suggest that considering the effects of genotype may be more necessary when the Mediterranean diet is not followed and that this diet may have a protective role against the effects of certain genotypes. Further studies are required to determine the potential mechanism of the observed gene-diet interaction.

**Clinical Trial Registration:**

[www.ClinicalTrials.gov], identifier [NCT04045392].

## Introduction

Obesity is a major public health problem worldwide and is associated with an increased risk of cancer recurrence and poor prognosis in patients with breast cancer (1, 2). As adipose tissue becomes the main source of estrogen biosynthesis after menopause (3), obese postmenopausal women have an especially high risk of adverse outcomes from breast cancer (4). Furthermore, recent studies have reported a significant association between obesity and poorer prognosis in hormone receptor-positive breast cancer (5, 6), and adjuvant hormone therapy, which is commonly used in hormone receptor-positive early disease, can cause side effects such as weight gain (7). Therefore, weight management is recommended in overweight or obese patients with hormone receptor-positive early breast cancer, especially if they are postmenopausal.

Weight management for overweight or obesity should include lifestyle modifications, such as dietary interventions and exercise (8). The Mediterranean diet, characterized by high consumption of vegetables, fruits, whole grains, legumes, nuts, and olive oil, as well as low intake of red or processed meat, has been proposed as an ideal healthy dietary pattern (9). Many studies have demonstrated the positive effects of the Mediterranean diet, not only for weight loss but also for a number of chronic diseases, such as cardiovascular disorders and various cancers (10, 11). Adherence to the Mediterranean diet has been consistently associated with a lower risk of breast cancer incidence and more favorable prognosis after diagnosis (12), suggesting potential beneficial effects of the Mediterranean diet for obese breast cancer patients.

In recent years, there has been increasing interest in the relationship between obesity-associated genes, such as the fat mass and obesity-associated (*FTO*), melanocortin-4 receptor (*MC4R*), transmembrane protein 18 (*TMEM18*), and brain-derived neurotrophic factor (*BDNF*) genes, and weight reduction or metabolic changes after behavioral interventions (13, 14). Studies have generally explored single nucleotide polymorphism (SNP) variants within well-documented genes—*FTO* and *MC4R* genes. The *FTO* gene is known to be firmly associated with higher body mass index and obesity risk (15), while the *MC4R* gene is the most common causative gene of monogenic obesity (16). Both *FTO* and *MC4R* are highly expressed in the hypothalamus, which regulates food intake and energy homeostasis (17, 18). Several studies have investigated associations between variations in these genes and a Mediterranean diet intervention (19–21). A sub-study of the PREDIMED trial showed that risk allele carriers of *FTO* rs9939609 had less weight gain than non-carriers after following the Mediterranean diet for 3 years, while no gene-diet interaction was found (20). In addition, Ortega-Azorín et al. reported significant gene-diet interactions of *FTO* rs9939609 and *MC4R* rs17782313 with adherence to the Mediterranean diet in type 2 diabetes (21). Identifying the relationships between genetic factors and nutrition could help suggest possible individualized nutritional solutions for prevention and management of metabolic diseases (22). However, the findings from previous studies remain controversial, and studies conducted in Asian populations are rare.

Thus, this pilot study was conducted to examine the influence of two obesity-associated SNP variants (*FTO* rs7185735 and *MC4R* rs476828) and the Mediterranean diet intervention on weight loss and modification of nutrient intake and metabolic parameters in overweight or obese, postmenopausal breast cancer patients receiving adjuvant hormone therapy.

## Materials and Methods

### Study Design and Participants

This 8-week, randomized, controlled dietary trial was performed at Gangnam Severance Hospital in Seoul, South Korea, from September 2019 to September 2020. The study design and experimental protocol were approved by the Institutional Review Board of Gangnam Severance Hospital (number 3-2019-0140), and the trial was registered at ClinicalTrials.gov (number NCT04045392) in August 2019. The study was conducted in accordance with the principles of the Declaration of Helsinki. All participants provided written informed consent before screening and data collection.

Study participants were recruited *via* poster and online advertisements at Gangnam Severance Hospital from August 2019 to June 2020. They were also recruited through direct referrals from breast surgeons. Inclusion criteria were as follows: breast cancer patients who were diagnosed with breast cancer stage I–III, who completed cancer treatment (surgery alone or surgery plus adjuvant chemotherapy or radiotherapy), and who are receiving adjuvant hormone therapy, postmenopausal, overweight or obese [body mass index (BMI) ≥ 23.0 kg/m^2^, according to the World Health Organization definition (23)]. Individuals who met any of the following criteria were excluded: cancer recurrence or metastasis, weight change more than 5 kg in the previous 3 months, secondary cause of obesity (such as hypothyroidism), hepatic or renal disease, serious psychiatric illness (such as bipolar disorder, schizophrenia, bulimia, anorexia nervosa, or suicidal ideation), current use of weight loss medications, receiving systemic corticosteroid therapy, history of alcohol abuse or dependence, or history of food allergy (such as seafood, fish, nuts, eggs, meat, tomatoes, or wheat).

### Randomization

Participants were randomly assigned to the Mediterranean diet (MeDiet) group or control group. Block randomization was performed using a computer-generated random number sequence with a block size of four. An independent statistician generated the allocation sequence, and the study coordinator assigned each participant to the intervention in chronologic order according to the enrollment date. Only outcome assessors were blinded to the group allocation.

### Dietary Intervention

Each group was instructed to follow their assigned diet protocol. All participants received dietary advice from research nutritionists at the first (baseline), second (week 4), and last (week 8) visits.

Participants in the MeDiet group received home delivery of two meals daily (breakfast and dinner) on 5 days per week during the 8-week intervention. Participants were advised to follow the Mediterranean diet, even for meals not delivered. The home delivery meals were prepared under the supervision of professional nutritionists by chefs trained on the principles of the Mediterranean diet. Each meal was restricted to 500 kcal, and the composition of nutrients was as follows: 50–55% of calories from carbohydrate, 20–25% of calories from protein, 30–35% of calories from fat, 8–11 g of fiber, and an omega-6:omega-3 ratio of 4–8:1. The composition of macronutrients was based on the results of the Dietary Intervention Randomized Controlled Trial (DIRECT) (24), as well as the macronutrient ratio with the lowest all-cause mortality identified in our previous study based on Korea National Health and Nutrition Examination Survey (KNHANS)–linked cause of death data (25). We used a ratio of omega-6:omega-3 of 4–8:1 rather than 4:1 in consideration of Korean dietary habits. In addition, two home delivery meals per day included 15 g of olive oil (3 servings), 1.5 servings of fruit, 4 servings of vegetables, 1 serving of nuts, and 3.5 serving of fish or meat. To assess adherence to the Mediterranean diet, we used the Korean version of the Mediterranean Diet Adherence Screener (K-MEDAS), which was developed and validated by our research team (26). However, the item for wine consumption was excluded because of negative evidence linking alcohol consumption with breast cancer. Consequently, K-MEDAS scores used in this study ranged from 0 to 13, with scores above 9 defined as high adherence to the Mediterranean diet.

The control group received only dietary advice based on recommendations of the 2015 Dietary Reference Intakes for Koreans (KDRI) (27). We aimed for an energy intake of 1,500 kcal per day with 55–65% of calories from carbohydrate, 7–20% of calories from protein, 15–30% calories from fat, 4–10% from omega-6, ≤ 1% from omega-3, and ≤ 300 mg of cholesterol per day.

### Genotyping

DNA was extracted from peripheral blood. All DNA samples were amplified and randomly cleaved into 25–125 bp fragments, which were then purified, resuspended, and hybridized with a Theragen Precision Medicine Research Array (Theragen PMRA), which is a customized array based on the Asian Precision Medicine Research Array (Thermo Fisher Scientific, Waltham, MA, United States). Following hybridization, the bound targets were washed under stringent conditions to remove non-specific background in order to minimize noise resulting from random ligation events. We genotyped 820,000 SNPs using the Theragen PMRA according to the manufacturer’s instructions, which provides genome-wide coverage in five major populations and imputation accuracies for genome-wide association study markers of 0.90 and 0.94 for minor allele frequencies > 1 and > 5%, respectively, for 7.4 million imputed markers in the Asian population. We analyzed two obesity-associated SNPs: *FTO* rs7185735 and *MC4R* rs476828. The subjects were classified as non-carriers or carriers: homozygous A (AA genotype) vs. G carriers for *FTO* rs7185735 and homozygous T (TT genotype) vs. C carriers for *MC4R* rs476828. The Hardy-Weinberg equilibrium was assessed for each SNP (Supplementary Table 1).

### Assessment of Outcomes

Body weight and composition were evaluated at each of the three visits. Body weight (to the nearest 0.1 kg) and height (to the nearest 0.1 cm) were measured using an automatic extensometer (BSM 330; Biospace, Seoul, South Korea) while patients were wearing light-weight clothing and no shoes. BMI was calculated as the ratio of weight (kg) to height squared (m^2^). Skeletal muscle mass, fat mass, and fat percentages were measured using a bioelectrical impedance analyzer (ACCUNIQ BC720; SELVAS Healthcare Inc., Daejeon, South Korea).

Participants were asked to complete self-reported questionnaires at baseline and after the intervention. The 24-h dietary recall method was used to calculate the total calorie intake and the composition of macro- and micronutrients. Adherence to the Mediterranean diet was evaluated using the aforementioned K-MEDAS, which included 13 items. The quantity of physical activity was calculated in metabolic equivalent-hours per week using the Godin Leisure-Time Exercise Questionnaire.

Fasting blood samples were collected from an antecubital vein to assess metabolic parameters at baseline and after the 8-week intervention. White blood cell counts were quantified with an XN-9000 hematology analyzer (Sysmex, IL, United States). Fasting glucose, high-sensitivity C-reactive protein, total cholesterol, triglycerides, high-density lipoprotein cholesterol, and low-density lipoprotein cholesterol (LDL-C) levels were measured with the ADVIA 1650 Clinical Chemistry system (Siemens Medical Solutions, Tarrytown, NY, United States). Fasting insulin was measured by an electrochemiluminescence immunoassay using an Elecsys 2010 instrument (Roche, Indianapolis, IN, United States). Insulin resistance was estimated using the homeostasis model assessment of insulin resistance (HOMA-IR) method by applying the following formula: HOMA-IR = fasting insulin (μIU/mL) × fasting glucose (mg/dL)/405 (28).

### Statistical Analysis

The normality of the distribution of variables was assessed using the Kolmogorov-Smirnov test. Data are presented as mean ± standard deviation (SD) or mean ± standard error of the mean (SEM). *T*-tests were used to compare baseline participant characteristics according to genotype. Changes in nutrient intake, body composition, and metabolic parameters were calculated by subtracting baseline measurements from measurements obtained at the end of the 8-week dietary intervention. Differences between the MeDiet and control groups, as well as differences between genotypes within each intervention group, were analyzed using the *t*-test or analysis of covariance adjusting for age and initial BMI. Gene-diet interactions on outcome changes were analyzed using a general linear regression model adjusting for age and initial BMI. *P* < 0.05 was considered statistically significant. All analyses were performed using SPSS for Windows (version 25.0; SPSS Inc., Chicago, IL, United States). All datasets used and/or analyzed during this study are available from the corresponding author upon reasonable request.

## Results

### Baseline Characteristics of Study Participants

**FIGURE 1:**
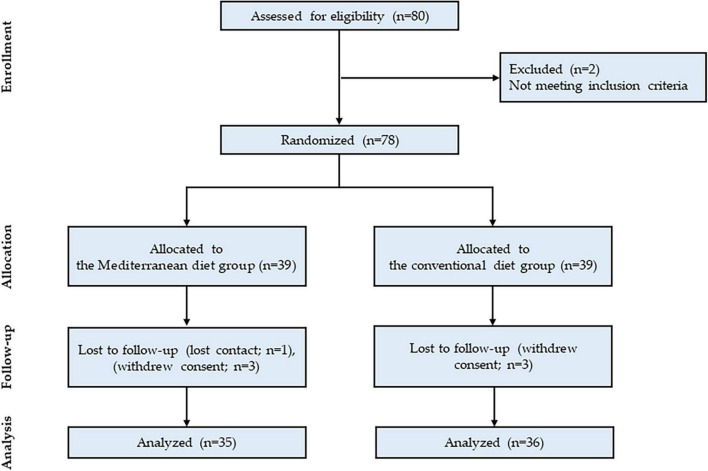
Flowchart of the study participant selection process.

A total of 80 candidates were screened, 78 of whom were enrolled and randomized to the study. The 8-week adherence rates were 89.7% in the MeDiet group and 92.3% in the control group (Chi-square test, *p* = 0.692) (Figure 1).

Baseline characteristics of study participants according to genotype are shown in Table 1. At baseline, there were no significant differences between non-carrier and carriers for age and body composition. Total cholesterol and LDL-C were significantly higher in C carriers than in patients with a TT genotype for *MC4R* rs476828 (*p* = 0.005 and *p* = 0.017, respectively). No other statistically significant differences in metabolic parameters were observed between genotypes at baseline.

**TABLE 1:** Baseline characteristics of study participants according to genotype.

	*FTO rs7185735*			*MC4R rs476828*		
	AA genotype	G carriers	*P-value*	TT genotype	C carriers	*P-value*
*n* (%)	48 (67.6)	23 (32.4)		35 (49.3)	36 (50.7)	
Age (y)	60.1 ± 6.2	60.7 ± 6.0	0.735	60.9 ± 6.4	59.8 ± 5.8	0.447
**Body composition**						
Weight (kg)	63.9 ± 6.1	63.2 ± 6.9	0.645	63.2 ± 5.4	64.2 ± 7.1	0.512
BMI (kg/m^2^)	26.3 ± 2.3	26.2 ± 2.4	0.822	26.3 ± 2.4	26.2 ± 2.3	0.875
Skeletal muscle (kg)	21.7 ± 2.7	20.7 ± 1.4	0.053	20.9 ± 2.0	21.8 ± 2.7	0.123
Fat mass (kg)	24.9 ± 4.2	25.5 ± 5.3	0.628	25.1 ± 3.7	25.0 ± 5.3	0.914
Fat percentage (%)	38.9 ± 4.6	39.9 ± 4.5	0.355	39.7 ± 3.9	38.7 ± 5.0	0.387
**Metabolic parameters**						
WBC count (no./μL)	5.50 ± 1.25	5.58 ± 1.72	0.841	5.44 ± 1.39	5.61 ± 1.44	0.600
hsCRP (mg/L)	1.0 ± 1.2	1.1 ± 1.0	0.787	1.2 ± 1.4	0.9 ± 0.9	0.381
Fasting glucose (mg/dL)	101.8 ± 10.6	101.3 ± 15.8	0.867	99.2 ± 12.7	104.0 ± 11.8	0.101
Insulin (μIU/mL)	10.4 ± 6.8	9.5 ± 5.4	0.590	9.9 ± 5.5	10.2 ± 7.1	0.840
HOMA-IR	2.68 ± 1.97	2.46 ± 1.63	0.639	2.49 ± 1.49	2.72 ± 2.18	0.604
TC (mg/dL)	192.0 ± 35.7	191.9 ± 40.9	0.993	179.6 ± 34.6	204.0 ± 36.1	0.005
Triglyceride (mg/dL)	141.5 ± 61.6	121.9 ± 45.2	0.179	131.7 ± 61.6	138.5 ± 53.3	0.624
HDL –C (mg/dL)	57.5 ± 10.3	59.6 ± 13.6	0.479	56.8 ± 10.5	59.6 ± 12.2	0.298
LDL-C (mg/dL)	111.0 ± 28.5	109.9 ± 30.5	0.881	102.4 ± 26.7	118.7 ± 29.1	0.017

*Data are expressed as mean ± SD. p-values were calculated using the t-test.*

*BMI, body mass index; HDL-C, high-density lipoprotein cholesterol; HOMA-IR, homeostasis model of assessment-insulin resistance; hsCRP, high-sensitivity C-reactive protein; LDL-C, low-density lipoprotein cholesterol; TC, total cholesterol; WBC, white blood cell.*

### Influence of Genotype and Dietary Intervention on Nutrient Intake and Energy Expenditure

Nutrient intake and energy expenditure at baseline, as well as changes after the intervention, are presented in Table 2. For the *FTO* rs7185735 variants, the MeDiet group exhibited the following significant differences, compared with the control group: greater decrease in carbohydrate intake (*p* = 0.023) and increase in protein intake (*p* = 0.012) in the AA genotype, greater decrease in trans fat intake (*p* = 0.006) in G carriers, and greater increase in monounsaturated fatty acid (MUFA) intake (*p* < 0.001 and *p* = 0.001, respectively) and K-MEDAS score (*p* < 0.001 for both) in both the AA genotype and G carriers. There were no significant differences in nutrient intake or energy expenditure between genotypes within the MeDiet group or within the control group. In addition, no significant interactions were found between *FTO* rs7185735 and dietary intervention for nutrient intake or energy expenditure (*p* > 0.05, Supplementary Table 2).

**TABLE 2:** Changes in nutrient intake and energy expenditure after intervention according to genotype.

*FTO rs7185735*								

	AA genotype			G carriers			MeDiet *P-value*^b^	Control *P-value*^c^
	MeDiet (*n* = 23)	Control (*n* = 25)	*P-value^a^*	MeDiet (*n* = 12)	Control (*n* = 11)	*P-value^a^*		
**Calorie (kcal/day)**								
Baseline	1257.5 ± 83.6	1449.8 ± 89.6	0.125	1350.1 ± 107.3	1445.7 ± 81.3	0.491	0.511	0.978
Change	−60.6 ± 98.8	−121.1 ± 92.6	0.657	−82.6 ± 114.1	−158.8 ± 126.8	0.659	0.891	0.819
**Carbohydrate (%)**								
Baseline	62.3 ± 2.5	58.8 ± 1.7	0.254	62.9 ± 3.0	61.1 ± 3.8	0.709	0.882	0.537
Change	−8.9 ± 2.7	−1.0 ± 2.0	0.023	−8.4 ± 3.0	−3.4 ± 3.5	0.288	0.918	0.539
**Protein (%)**								
Baseline	16.0 ± 1.0	16.9 ± 1.0	0.494	15.5 ± 1.4	15.6 ± 1.2	0.942	0.796	0.438
Change	2.9 ± 1.2	−1.0 ± 0.9	0.012	3.0 ± 1.4	−0.1 ± 1.0	0.094	0.964	0.570
**Total fat (%)**								
Baseline	22.2 ± 1.8	23.9 ± 1.4	0.463	22.3 ± 2.3	22.9 ± 3.6	0.876	0.991	0.762
Change	7.4 ± 1.8	2.6 ± 1.9	0.078	6.3 ± 2.7	3.5 ± 3.0	0.486	0.753	0.808
**SFA (%)**								
Baseline	5.3 ± 0.8	5.7 ± 0.7	0.676	5.1 ± 0.7	5.0 ± 0.7	0.871	0.886	0.517
Change	0.5 ± 0.9	0.5 ± 1.1	0.983	−0.1 ± 0.9	1.6 ± 0.9	0.204	0.696	0.495
**MUFA (%)**								
Baseline	5.8 ± 0.7	5.3 ± 0.6	0.592	5.5 ± 0.9	5.2 ± 0.7	0.779	0.783	0.886
Change	8.1 ± 1.0	0.9 ± 1.1	<0.001	8.7 ± 1.6	1.5 ± 1.0	0.001	0.724	0.733
**PUFA (%)**								
Baseline	4.4 ± 0.5	4.7 ± 0.5	0.621	4.9 ± 0.8	4.3 ± 0.6	0.550	0.609	0.551
Change	2.1 ± 0.8	0.1 ± 0.7	0.057	2.8 ± 1.2	1.6 ± 0.7	0.420	0.585	0.190
**Trans fat (%)**								
Baseline	0.22 ± 0.03	0.26 ± 0.04	0.376	0.23 ± 0.05	0.20 ± 0.05	0.640	0.843	0.327
Change	−0.08 ± 0.04	0.06 ± 0.07	0.096	−0.14 ± 0.06	0.13 ± 0.06	0.006	0.436	0.557
**K-MEDAS score**								
Baseline	6.0 ± 0.4	6.0 ± 0.4	1.000	6.7 ± 0.5	5.6 ± 0.4	0.136	0.314	0.611
Change	6.2 ± 0.4	0.4 ± 0.3	<0.001	5.9 ± 0.5	0.9 ± 0.5	<0.001	0.672	0.401
**GLTEQ score**								
Baseline	30.3 ± 6.7	17.5 ± 3.6	0.099	22.5 ± 5.6	13.3 ± 3.7	0.191	0.377	0.489
Change	−6.3 ± 6.4	3.7 ± 3.0	0.167	6.8 ± 7.4	16.1 ± 9.7	0.450	0.192	0.246

***MC4R rs476828***								

	**TT genotype**			**C carriers**			**MeDiet** ***p***-**value^b^**	**Control** ***p***-**value^c^**
	**MeDiet** **(n = 17)**	**Control** **(n = 18)**	***P-value* ^a^**	**MeDiet** **(n = 18)**	**Control** **(n = 18)**	***P-value* ^a^**		

**Calorie (kcal/day)**								
Baseline	1272.1 ± 97.6	1539.5 ± 96.5	0.060	1305.4 ± 90.8	1357.6 ± 88.6	0.683	0.804	0.174
Change	−7.4 ± 113.4	−150.2 ± 99.8	0.350	−125.5 ± 99.7	−115.1 ± 112.4	0.945	0.438	0.817
**Carbohydrate (%)**								
Baseline	62.7 ± 2.4	59.0 ± 2.5	0.290	62.3 ± 3.0	60.1 ± 2.3	0.551	0.925	0.754
Change	−9.1 ± 2.4	−1.0 ± 2.6	0.027	−8.3 ± 3.3	−2.6 ± 2.4	0.169	0.852	0.638
**Protein (%)**								
Baseline	15.8 ± 1.1	16.3 ± 0.8	0.727	15.8 ± 1.3	16.8 ± 1.3	0.589	0.978	0.744
Change	3.2 ± 1.1	0.0 ± 0.8	0.028	2.7 ± 1.4	−1.4 ± 1.2	0.031	0.805	0.325
**Total fat (%)**								
Baseline	21.5 ± 1.4	24.4 ± 2.0	0.227	22.9 ± 2.4	22.7 ± 2.2	0.945	0.595	0.559
Change	7.8 ± 1.6	1.0 ± 2.0	0.012	6.3 ± 2.5	4.7 ± 2.4	0.662	0.622	0.245
**SFA (%)**								
Baseline	4.7 ± 0.5	6.7 ± 0.8	0.057	5.8 ± 0.9	4.4 ± 0.6	0.211	0.342	0.028
Change	0.9 ± 0.6	−1.5 ± 1.0	0.050	−0.2 ± 1.2	3.1 ± 1.0	0.037	0.401	0.002
**MUFA (%)**								
Baseline	4.9 ± 0.4	6.1 ± 0.7	0.141	6.5 ± 1.0	4.5 ± 0.6	0.089	0.145	0.083
Change	9.0 ± 0.9	−0.3 ± 1.0	<0.001	7.6 ± 1.4	2.4 ± 1.2	0.008	0.439	0.098
**PUFA (%)**								
Baseline	4.5 ± 0.5	4.3 ± 0.4	0.732	4.6 ± 0.6	4.9 ± 0.6	0.764	0.929	0.457
Change	2.4 ± 0.6	1.3 ± 0.8	0.242	2.2 ± 1.1	−0.2 ± 0.8	0.082	0.835	0.185
**Trans fat (%)**								
Baseline	0.21 ± 0.04	0.28 ± 0.05	0.023	0.23 ± 0.04	0.21 ± 0.04	0.003	0.953	0.044
Change	−0.08 ± 0.04	−0.04 ± 0.07	0.560	−0.11 ± 0.06	0.21 ± 0.06	0.001	0.713	0.016
**K-MEDAS score**								
Baseline	6.2 ± 0.5	5.6 ± 0.4	0.341	6.3 ± 0.4	6.2 ± 0.5	0.931	0.873	0.309
Change	6.4 ± 0.5	0.6 ± 0.4	<0.001	5.8 ± 0.3	0.6 ± 0.4	<0.001	0.365	1.000
**GLTEQ score**								
Baseline	27.2 ± 8.0	13.6 ± 3.3	0.128	28.0 ± 5.6	18.9 ± 4.3	0.206	0.934	0.334
Change	3.8 ± 8.1	12.0 ± 5.6	0.404	−7.0 ± 5.8	3.0 ± 4.6	0.187	0.282	0.222

*Data are expressed as mean ± SEM. P-values were calculated using the t-test.*

*^a^Differences between Mediterranean diet and conventional diet groups.*

*^b^Differences between genotypes in the Mediterranean diet group.*

*^c^Differences between genotypes in the conventional diet group.*

*GLTEQ, Godin Leisure-Time Exercise Questionnaire; K-MEDAS, Korean version of the Mediterranean Diet Adherence Screener; MeDiet, Mediterranean diet; MUFA, monounsaturated fatty acid; PUFA, polyunsaturated fatty acid; SFA, saturated fatty acid.*

**FIGURE 2:**
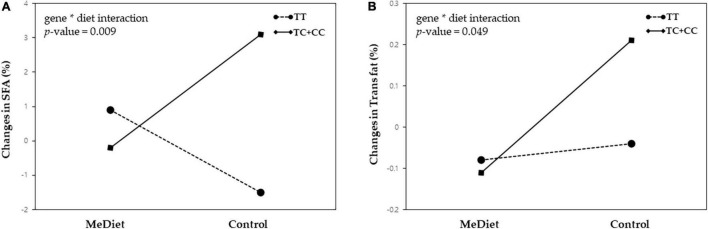
Gene-diet interactions between the MC4R rs476828 variant and dietary intervention for changes in **(A)** saturated fatty acid (SFA) intake and **(B)** trans fat intake.

For the *MC4R* rs476828 variants, the MeDiet group exhibited the following significant differences, compared with the control group: greater decrease in carbohydrate intake (*p* = 0.027) and increase in total fat intake (*p* = 0.012) in the TT genotype; greater decrease in saturated fatty acid (SFA) intake (*p* = 0.037) and trans fat intake (*p* = 0.001) in C carriers; and greater increase in protein intake (*p* = 0.028 and *p* = 0.031, respectively), MUFA intake (*p* < 0.001 and *p* = 0.008, respectively), and K-MEDAS score (*p* < 0.001 for both) in both the TT genotype and C carriers. Baseline values and changes in SFA and trans fat intake exhibited statistically significant differences between the TT genotype and C carriers only in the control group. Baseline intake of both SFA and trans fat were higher in the TT genotype than in C carriers (*p* = 0.028 and *p* = 0.044, respectively); however, after 8 weeks, intake of SFA and trans fat decreased in the TT genotype and increased in C carriers (*p* = 0.002 and *p* = 0.016 for SFA and trans fat, respectively). Furthermore, we found significant interactions between *MC4R* rs476828 and dietary intervention for changes in SFA intake (*p* = 0.009) and trans fat intake (*p* = 0.049), after adjusting for age and initial BMI (Figure 2).

### Influence of Genotype and Dietary Intervention on Body Composition and Metabolic Parameters

Table 3 shows the changes in body composition and metabolic parameters after the intervention. For the *FTO* rs7185735 variants, the MeDiet group exhibited the following significant differences, compared with the control group, after adjusting for age and initial BMI: greater decrease in weight (*p* < 0.001 and *p* = 0.011, respectively) and BMI (*p* < 0.001 and *p* = 0.011, respectively) in both the AA genotype and G carriers; greater decrease in fat mass (*p* = 0.002) and fat percentage (*p* = 0.022) in the AA genotype; and greater decrease in insulin (*p* = 0.004), HOMA-IR (*p* = 0.015), and triglycerides (*p* = 0.15) in G carriers. We found no significant differences for changes in body composition or metabolic parameters between genotypes within the MeDiet group or within the control group. No significant gene-diet interactions were found between *FTO* rs7185735 and dietary intervention (*p* > 0.05, Supplementary Table 3).

**TABLE 3:** Changes in body composition and metabolic parameters after intervention according to genotype.

*FTO rs7185735*								

	AA genotype			G carriers			MeDiet *P-value*^b^	Control *P-value*^c^
	MeDiet (*n* = 23)	Control (*n* = 25)	*P-value* ^a^	MeDiet (*n* = 12)	Control (*n* = 11)	*P-value* ^a^		
Weight (kg)	−2.7 ± 0.4	−0.5 ± 0.3	<0.001	−2.9 ± 0.6	−0.3 ± 0.6	0.011	0.974	0.787
BMI (kg/m^2^)	−1.2 ± 0.2	−0.2 ± 0.1	<0.001	−1.2 ± 0.2	−0.1 ± 0.2	0.011	0.870	0.762
Skeletal muscle (kg)	−0.8 ± 0.4	−0.4 ± 0.2	0.286	−0.9 ± 0.2	0.1 ± 0.2	0.011	0.920	0.471
Fat mass (kg)	−2.0 ± 0.5	0.2 ± 0.4	0.002	−1.3 ± 0.5	−0.4 ± 0.6	0.234	0.394	0.656
Fat percentage (%)	−1.6 ± 0.7	0.4 ± 0.5	0.022	−0.1 ± 0.4	−0.4 ± 0.7	0.875	0.186	0.557
WBC count (no./μL)	−0.57 ± 0.27	−0.19 ± 0.24	0.283	−0.39 ± 0.33	−0.24 ± 0.34	0.958	0.705	0.672
hsCRP (mg/L)	1.3 ± 1.3	0.6 ± 0.6	0.663	0.2 ± 0.5	−0.1 ± 0.3	0.453	0.631	0.421
Fasting glucose (mg/dL)	2.4 ± 1.6	−0.1 ± 2.2	0.373	−2.0 ± 3.3	−0.4 ± 2.8	0.700	0.235	0.856
Insulin (μIU/mL)	−1.8 ± 0.8	−0.8 ± 1.2	0.551	−2.9 ± 1.3	0.5 ± 0.8	0.004	0.476	0.462
HOMA-IR	−0.38 ± 0.20	−0.23 ± 0.43	0.774	−0.72 ± 0.35	0.02 ± 0.25	0.015	0.407	0.669
TC (mg/dL)	−9.7 ± 4.2	−7.3 ± 3.7	0.729	−7.1 ± 4.9	−6.0 ± 7.5	0.623	0.839	0.592
Triglycerides (mg/dL)	−33.4 ± 14.3	5.0 ± 14.3	0.060	−28.6 ± 9.2	15.6 ± 13.4	0.015	0.802	0.722
HDL-C (mg/dL)	−0.8 ± 1.3	−3.9 ± 1.8	0.185	−1.3 ± 2.5	0.4 ± 1.9	0.609	0.764	0.197
LDL-C (mg/dL)	−7.5 ± 2.7	−5.2 ± 2.8	0.624	−4.3 ± 4.0	-8.0 ± 5.2	0.819	0.633	0.869

***MC4R rs476828***								

	**TT genotype**			**C carriers**			**MeDiet** ***P-value*^b^**	**Control** ***P-value*^c^**
	**MeDiet** **(*n* = 17)**	**Control** **(*n* = 18)**	***P-value* ^a^**	**MeDiet** **(*n* = 18)**	**Control** **(*n* = 18)**	***P-value* ^a^**		

Weight (kg)	−2.5 ± 0.4	−0.3 ± 0.4	0.001	−3.1 ± 0.5	−0.5 ± 0.4	0.001	0.323	0.785
BMI (kg/m^2^)	−1.1 ± 0.2	−0.1 ± 0.2	0.001	−1.3 ± 0.2	−0.2 ± 0.2	0.001	0.417	0.776
Skeletal muscle (kg)	−0.4 ± 0.2	0.0 ± 0.1	0.066	−1.2 ± 0.5	−0.5 ± 0.3	0.298	0.119	0.082
Fat mass (kg)	−1.8 ± 0.5	−0.4 ± 0.4	0.029	−1.7 ± 0.6	0.4 ± 0.5	0.005	0.750	0.228
Fat percentage (%)	−1.4 ± 0.7	−0.4 ± 0.4	0.265	−0.8 ± 0.8	0.8 ± 0.7	0.051	0.960	0.090
WBC count (no./μL)	−0.67 ± 0.26	−0.53 ± 0.28	0.713	−0.36 ± 0.33	0.12 ± 0.24	0.284	0.534	0.092
hsCRP (mg/L)	−0.4 ± 0.2	0.2 ± 0.5	0.251	2.1 ± 1.6	0.7 ± 0.7	0.277	0.057	0.575
Fasting glucose (mg/dL)	3.9 ± 1.9	−0.3 ± 2.6	0.224	−2.0 ± 2.3	−0.1 ± 2.3	0.467	0.028	0.972
Insulin (μIU/mL)	−2.8 ± 1.2	−0.5 ± 1.2	0.225	−1.5 ± 0.7	−0.4 ± 1.3	0.600	0.584	0.974
HOMA-IR	−0.60 ± 0.30	−0.22 ± 0.35	0.484	−0.40 ± 0.19	−0.09 ± 0.51	0.712	0.866	0.890
TC (mg/dL)	−9.1 ± 5.2	−10.8 ± 5.5	0.773	−8.5 ± 3.9	−2.9 ± 3.9	0.168	0.647	0.207
Triglycerides (mg/dL)	−46.4 ± 15.8	5.3 ± 14.5	0.031	−17.9 ± 11.5	11.2 ± 16.0	0.176	0.253	0.779
HDL-C (mg/dL)	0.9 ± 1.7	−2.9 ± 1.4	0.096	−2.7 ± 1.6	−2.3 ± 2.5	0.936	0.211	0.846
LDL-C (mg/dL)	−7.3 ± 3.8	−9.2 ± 3.6	0.652	−5.6 ± 2.6	−2.9 ± 3.3	0.341	0.853	0.165

*Data are expressed as mean ± SEM. p-values were calculated using analysis of covariance, adjusted for age and initial BMI.*

*^a^Differences between Mediterranean diet and conventional diet groups.*

*^b^Differences among genotypes in Mediterranean diet group.*

*^c^Differences among genotypes in conventional diet group.*

*BMI, body mass index; hsCRP, high-sensitivity C-reactive protein; HDL-C, high-density lipoprotein cholesterol; HOMA-IR, homeostasis model of assessment-insulin resistance; LDL-C, low-density lipoprotein cholesterol; MeDiet, Mediterranean diet; TC, total cholesterol; WBC, white blood cell.*

For the *MC4R* rs476828 variants, the MeDiet group exhibited the following significant differences, compared with the control group, after adjusting for age and initial BMI: greater decrease in weight (*p* = 0.001 for both), BMI (*p* = 0.001 for both), fat mass (*p* = 0.029 and *p* = 0.005, respectively) in both the TT genotype and C carriers; and greater decrease in triglycerides (*p* = 0.031) in the TT genotype. Only changes in fasting glucose differed significantly between the TT genotype and C carriers in the MeDiet group (*p* = 0.028). However, no significant interaction was found between *MC4R* rs476828 and dietary intervention for changes in body composition or metabolic parameters (*p* > 0.05, Supplementary Table 3).

## Discussion

In this study, we examined the influence of obesity-associated SNPs and Mediterranean diet on body composition, metabolic parameters, and nutrient intake in overweight or obese postmenopausal women with breast cancer receiving adjuvant hormone therapy. Our results showed that *FTO* rs7185735 and *MC4R* rs476828 variants did not influence weight loss or improvement of metabolic parameters in patients receiving the Mediterranean diet intervention. SFA and trans fat intake were influenced by the *MC4R* rs476828 variant, with both SFA and trans fat significantly increased in C carriers, compared with the TT genotype, although only in the control group. There were also statistically significant interactions between *MC4R* rs476828 and dietary intervention for changes in both SFA and trans fat intake.

The Mediterranean diet has been proposed as one of the healthiest dietary patterns worldwide. Although the duration of dietary intervention was short, our results are consistent with those of previous clinical trials demonstrating beneficial effects of the short-term Mediterranean dietary intervention for weight loss and cardiometabolic risk improvement (29, 30). We found that body weight, BMI, fat mass, and fat percentage were significantly decreased in the MeDiet group compared to the control group after adjusting for age and initial BMI (Supplementary Table 4). Among metabolic parameters, only triglycerides significantly decreased in the MeDiet group compared to the control group. The MeDiet group had greater weight loss and metabolic improvement with no significant change in caloric intake (Supplementary Table 5). Several previous studies have also found that calorie-unrestricted Mediterranean diet intervention reduces weight and cardiometabolic risk, supporting the importance of diet quality as well as quantity (29, 31). With its emphasis on plant-based foods, the Mediterranean diet contains a high amount of fiber, low energy density, and low glycemic load, which is a major contributor to its weight-loss effects (32). MUFA from olive oil and omega-3 fatty acids from nuts and fish, both of which are important components of the Mediterranean diet, have been shown to reduce serum triglycerides by suppressing the postprandial increase in triglycerides, enhancing triglyceride clearance, and decreasing overall synthesis of triglycerides (33, 34). Although direct effects of the Mediterranean diet on breast cancer were not examined in this study, improvements in obesity and metabolic parameters are believed to improve the prognosis of patients with early stage breast cancer.

In this study, we also investigated the influence of two representative obesity-associated genes (*FTO* and *MC4R*) on body composition, metabolic parameters, and nutrient intake, as well as changes in these factors after intervention within the MeDiet group and control group. At baseline, only total cholesterol and LDL-C were significantly higher in risk allele carriers of the *MC4R* rs476828 variant, unlike previous studies reporting higher BMI and waist circumferences in risk allele carriers of both *FTO* and *MC4R* variants (35, 36). This discrepancy may reflect the methodology of the current pilot study, which included a smaller number of participants and only overweight or obese patients, although we did analyze two SNPs found by our research team to be highly associated with obesity in the Korean population (unpublished data).

According to our results, there were no significant interactions between obesity-associated SNP variants and dietary intervention for changes in body composition or metabolic parameters, which is consistent with the results of several previous studies (37, 38). By contrast, there were significant interactions between *MC4R* rs476828 and dietary intervention for changes in both SFA intake and trans fat intake. Di Renzo et al. (39) reported a significant gene-diet interaction only for total body fat mass among body composition parameters and suggested that this finding may reflect the considerable total body fat mass gained by risk allele carriers of the *FTO* variant in the control group. Ortega-Azorín et al. (21) showed that risk allele carriers of *FTO* and *MC4R* variants had a higher risk of type 2 diabetes when adherence to the Mediterranean diet was low; however, these associations disappeared when adherence was high. Similarly, we found that risk allele carriers of *MC4R* rs476828 had a significantly greater increase in SFA and trans fat intake (compared with the TT genotype group) after the 8-week intervention only in the control group, whereas no significant differences were found between genotypes in the MeDiet group.

The precise mechanism whereby only the *MC4R* variant exhibited significant gene-diet interactions for changes in SFA and trans fat intake is unclear. Our findings are in line with the results of previous experimental and observational studies demonstrating that *MC4R* plays a role in controlling fat consumption (40–42). *MC4R* knockout mice exhibited severe high fat–induced hyperphagia (40), and injection of an *MC4R* agonist into the amygdala of mice reduced preference for a high-fat diet (41). Furthermore, the prospective cohort study of Qi et al. confirmed a significant association between *MC4R* rs17782313 and high intake of dietary fat (42). Nevertheless, it is difficult to explain the differences in *FTO* and *MC4R* effects observed in our study.

The lack of significant differences between genotypes in the MeDiet group may be attributed to a protective role of the Mediterranean diet. Previous nutrigenomic studies have shown that the Mediterranean diet reduces the expression of genes related to oxidative stress, inflammation, and atherogenesis (43, 44). Furthermore, anti-inflammatory effects of the nutrients and bioactive food components of the Mediterranean diet have been associated with hypermethylation of pro-inflammatory genes (45). Although the direct effects of the Mediterranean diet on *FTO* and *MC4R* remain unknown, the Mediterranean diet may modulate the effects of these genes through epigenetic mechanisms since both *FTO* (46) and *MC4R* (47) genes are regulated by methylation.

Increased intake of unhealthy fats, such as SFA and trans fat, another potential risk factor for breast cancer (48), was significant only in risk allele carriers of the *MC4R* variant in the control group, whereas significance was not observed in the MeDiet group. This finding suggests that considering the role of genotypes may be most important when a healthy (Mediterranean) diet is not followed.

Our study had some limitations. First, this pilot study included a relatively small number of participants. Second, the 8-week intervention may not have been long enough to investigate sufficient changes in metabolic parameters, and could not confirm the prognosis or recurrence of breast cancer. This is a preliminary pilot study for the future studies, and further randomized controlled trials with a longer intervention period and larger sample size are required to determine the effects of SNPs and Mediterranean diet on cardiometabolic risk and breast cancer prognosis. Third, nutrient intake was analyzed using the 24-h dietary recall method, which may have led to recall bias. However, the changes in nutrient intake in the MeDiet group were consistent with the composition of the Mediterranean diet. Fourth, body composition was measured using a bioelectrical impedance analyzer instead of more reliable methods, such as dual-energy x-ray absorptiometry (49). Nevertheless, bioelectrical impedance analysis is also a validated methodology, with the advantages of low cost and non-invasiveness. Finally, since only one SNP in each of the *FTO* and *MC4R* genes was analyzed, we could not determine the influence of other potential genetic variants in these or other genes. Further studies are required to explore associations between the Mediterranen diet and various other genetic variants including *FTO* rs9939609, *MC4R* rs17782313, *TMEM18* rs939583, and *BDNF* rs16917237.

The main strength and novelty of our study is that, to the best of our knowledge, we are the first to examine the association between two obesity-associated SNPs and the Mediterranean diet intervention in breast cancer patients. Furthermore, although this is a pilot study, it is a randomized controlled trial with a control group, and the MeDiet group was provided with home-delivered meals prepared according to Mediterranean diet principles for more precise dietary intervention. Third, we used the validated K-MEDAS questionnaire to assess adherence to the Mediterranean diet.

## Conclusion

In summary, we found that the *FTO* rs7185735 and *MC4R* rs476828 variants did not influence weight loss or improvement of metabolic parameters within the Mediterranean diet intervention in overweight or obese postmenopausal women with breast cancer receiving adjuvant hormone therapy. Among nutrient intake parameters, only changes in SFA and trans fat intake were influenced by the *MC4R* rs476828 variant in the control group and showed a significant gene-diet interaction. Further studies are necessary to investigate gene-diet interactions between nutrient and bioactive food components of the Mediterranean diet and various other genes, as well as the mechanisms of any observed interactions.

## Data Availability Statement

The original contributions presented in this study are included in the article/Supplementary Material, further inquiries can be directed to the corresponding author/s.

## Ethics Statement

The studies involving human participants were reviewed and approved by the Institutional Review Board of Gangnam Severance Hospital. The patients/participants provided their written informed consent to participate in this study.

## Author Contributions

A-RC, K-WH, Y-JK, H-MK, SB, SA, JJ, and J-WL: conceptualization and investigation. A-RC, K-WH, J-EC, and H-SL: analysis and interpretation of data. A-RC: writing—original draft preparation. K-WH, JJ, and J-WL: writing—review and editing. All authors have read and agreed to the published version of the manuscript.

## Conflict of Interest

K-WH and J-EC were employed by Theragen Etex Bio Institute Co., Ltd. The remaining authors declare that the research was conducted in the absence of any commercial or financial relationships that could be construed as a potential conflict of interest.

## Publisher’s Note

All claims expressed in this article are solely those of the authors and do not necessarily represent those of their affiliated organizations, or those of the publisher, the editors and the reviewers. Any product that may be evaluated in this article, or claim that may be made by its manufacturer, is not guaranteed or endorsed by the publisher.
