# Supplementary material for: Effects of Single Nucleotide Polymorphisms and Mediterranean Diet in Overweight or Obese Postmenopausal Women With Breast Cancer Receiving Adjuvant Hormone Therapy: A Pilot Randomized Controlled Trial
Source: Front Nutr. 2022 Jul 1;9:882717. doi: 10.3389/fnut.2022.882717 (PMC9284001; doi:10.3389/fnut.2022.882717)
Supplement: Supplementary file 1 [file Table_1.DOCX]

**Table S1** The Hardy-Weinberg equilibrium analyses for each SNP.

| **Genotype** |  | **Actual values** |  | **Expected Value** |  | **χ2** |  | ***p*-value** |
| --- | --- | --- | --- | --- | --- | --- | --- | --- |
| FTO rs7185735 | | | | | | | | |
| AA |  | 48 |  | 48.20 |  | 0.043 |  | 0.836 |
| AG |  | 21 |  | 20.60 |  |  |  |  |
| GG |  | 2 |  | 2.20 |  |  |  |  |
| MC4R rs476828 | | | | | | | | |
| TT |  | 35 |  | 34.51 |  | 0.008 |  | 0.930 |
| TC |  | 29 |  | 29.98 |  |  |  |  |
| CC |  | 7 |  | 6.51 |  |  |  |  |
