# Supplementary material for: Effects of Single Nucleotide Polymorphisms and Mediterranean Diet in Overweight or Obese Postmenopausal Women With Breast Cancer Receiving Adjuvant Hormone Therapy: A Pilot Randomized Controlled Trial
Source: Front Nutr. 2022 Jul 1;9:882717. doi: 10.3389/fnut.2022.882717 (PMC9284001; doi:10.3389/fnut.2022.882717)
Supplement: Supplementary file 2 [file Table_2.DOCX]

**Table S2** Gene-diet interactions between *FTO* rs7185735, *MC4R* rs476828 variants and dietary intervention for changes in nutrient intake and energy expenditure.

| ***FTO rs7185735*** | | | | | | |
| --- | --- | --- | --- | --- | --- | --- |
|  | **AA genotype** | |  | **G carriers** | | **gene * diet**  **interaction**  ***p*-value** |
|  | **MeDiet**  **(n=23)** | **Control**  **(n=25)** |  | **MeDiet**  **(n=12)** | **Control**  **(n=11)** |  |
| Calorie (kcal/day) | -60.6 ± 98.8 | -121.1 ± 92.6 |  | -82.6 ± 114.1 | -158.8±126.8 | 0.968 |
| Carbohydrate (%) | -8.9 ± 2.7 | -1.0 ± 2.0 |  | -8.4 ± 3.0 | -3.4 ± 3.5 | 0.635 |
| Protein (%) | 2.9 ± 1.2 | -1.0 ± 0.9 |  | 3.0 ± 1.4 | -0.1 ± 1.0 | 0.852 |
| Total fat (%) | 7.4 ± 1.8 | 2.6 ± 1.9 |  | 6.3 ± 2.7 | 3.5 ± 3.0 | 0.620 |
| SFA (%) | 0.5 ± 0.9 | 0.5 ± 1.1 |  | -0.1 ± 0.9 | 1.6 ± 0.9 | 0.510 |
| MUFA (%) | 8.1 ± 1.0 | 0.9 ± 1.1 |  | 8.7 ± 1.6 | 1.5 ± 1.0 | 0.948 |
| PUFA (%) | 2.1 ± 0.8 | 0.1 ± 0.7 |  | 2.8 ± 1.2 | 1.6 ± 0.7 | 0.501 |
| Trans fat (%) | -0.08 ± 0.04 | 0.06 ± 0.07 |  | -0.14 ± 0.06 | 0.13 ± 0.06 | 0.440 |
| K-MEDAS score | 6.2 ± 0.4 | 0.4 ± 0.3 |  | 5.9 ± 0.5 | 0.9 ± 0.5 | 0.370 |
| GLTEQ score | -6.3 ± 6.4 | 3.7 ± 3.0 |  | 6.8 ± 7.4 | 16.1 ± 9.7 | 0.855 |
| ***MC4R rs476828*** | | | | | | |
|  | **TT genotype** | |  | **C carriers** | | **gene * diet**  **interaction**  ***p*-value** |
|  | **MeDiet**  **(n=17)** | **Control**  **(n=18)** |  | **MeDiet**  **(n=18)** | **Control**  **(n=18)** |  |
| Calorie (kcal/day) | -7.4 ± 113.4 | -150.2 ± 99.8 |  | -125.5 ± 99.7 | -115.1±112.4 | 0.483 |
| Carbohydrate (%) | -9.1 ± 2.4 | -1.0 ± 2.6 |  | -8.3 ± 3.3 | -2.6 ± 2.4 | 0.812 |
| Protein (%) | 3.2 ± 1.1 | 0.0 ± 0.8 |  | 2.7 ± 1.4 | -1.4 ± 1.2 | 0.597 |
| Total fat (%) | 7.8 ± 1.6 | 1.0 ± 2.0 |  | 6.3 ± 2.5 | 4.7 ± 2.4 | 0.328 |
| SFA (%) | 0.9 ± 0.6 | -1.5 ± 1.0 |  | -0.2 ± 1.2 | 3.1 ± 1.0 | 0.009 |
| MUFA (%) | 9.0 ± 0.9 | -0.3 ± 1.0 |  | 7.6 ± 1.4 | 2.4 ± 1.2 | 0.119 |
| PUFA (%) | 2.4 ± 0.6 | 1.3 ± 0.8 |  | 2.2 ± 1.1 | -0.2 ± 0.8 | 0.487 |
| Trans fat (%) | -0.08 ± 0.04 | -0.04 ± 0.07 |  | -0.11 ± 0.06 | 0.21 ± 0.06 | 0.049 |
| K-MEDAS score | 6.4 ± 0.5 | 0.6 ± 0.4 |  | 5.8 ± 0.3 | 0.6 ± 0.4 | 0.399 |
| GLTEQ score | 3.8 ± 8.1 | 12.0 ± 5.6 |  | -7.0 ± 5.8 | 3.0 ± 4.6 | 0.937 |

Data are expressed as mean ± SEM. Gene-diet interaction *p*-values are calculated using a general linear regression model adjusting for age and initial BMI. Abbreviations: GLTEQ, Godin Leisure-Time Exercise Questionnaire; K-MEDAS, Korean version of the Mediterranean Diet Adherence Screener; MeDiet, Mediterranean diet; MUFA, monounsaturated fatty acid; PUFA, polyunsaturated fatty acid; SFA, saturated fatty acid.
