# Supplementary material for: Effects of Single Nucleotide Polymorphisms and Mediterranean Diet in Overweight or Obese Postmenopausal Women With Breast Cancer Receiving Adjuvant Hormone Therapy: A Pilot Randomized Controlled Trial
Source: Front Nutr. 2022 Jul 1;9:882717. doi: 10.3389/fnut.2022.882717 (PMC9284001; doi:10.3389/fnut.2022.882717)
Supplement: Supplementary file 3 [file Table_3.DOCX]

**Table S3** Gene-diet interactions between *FTO* rs7185735, *MC4R* rs476828 variants and dietary intervention for changes in body composition and metabolic parameters.

| ***FTO rs7185735*** | | | | | | |
| --- | --- | --- | --- | --- | --- | --- |
|  | **AA genotype** | |  | **G carriers** | | **gene * diet**  **interaction**  ***p*-value** |
|  | **MeDiet**  **(n=23)** | **Control**  **(n=25)** |  | **MeDiet**  **(n=12)** | **Control**  **(n=11)** |  |
| Weight (kg) | -2.7 ± 0.4 | -0.5 ± 0.3 |  | -2.9 ± 0.6 | -0.3 ± 0.6 | 0.840 |
| BMI (kg/m^2^) | -1.2 ± 0.2 | -0.2 ± 0.1 |  | -1.2 ± 0.2 | -0.1 ± 0.2 | 0.937 |
| Skeletal muscle (kg) | -0.8 ± 0.4 | -0.4 ± 0.2 |  | -0.9 ± 0.2 | 0.1 ± 0.2 | 0.764 |
| Fat mass (kg) | -2.0 ± 0.5 | 0.2 ± 0.4 |  | -1.3 ± 0.5 | -0.4 ± 0.6 | 0.352 |
| Fat percentage (%) | -1.6 ± 0.7 | 0.4 ± 0.5 |  | -0.1 ± 0.4 | -0.4 ± 0.7 | 0.160 |
| WBC count (no./μL) | -0.57 ± 0.27 | -0.19 ± 0.24 |  | -0.39 ± 0.33 | -0.24 ± 0.34 | 0.581 |
| hsCRP (mg/L) | 1.3 ± 1.3 | 0.6 ± 0.6 |  | 0.2 ± 0.5 | -0.1 ± 0.3 | 0.993 |
| Fasting glucose (mg/dL) | 2.4 ± 1.6 | -0.1 ± 2.2 |  | -2.0 ± 3.3 | -0.4 ± 2.8 | 0.517 |
| Insulin (μIU/mL) | -1.8 ± 0.8 | -0.8 ± 1.2 |  | -2.9 ± 1.3 | 0.5 ± 0.8 | 0.304 |
| HOMA-IR | -0.38 ± 0.20 | -0.23 ± 0.43 |  | -0.72 ± 0.35 | 0.02 ± 0.25 | 0.431 |
| TC (mg/dL) | -9.7 ± 4.2 | -7.3 ± 3.7 |  | -7.1 ± 4.9 | -6.0 ± 7.5 | 0.776 |
| Triglycerides (mg/dL) | -33.4 ± 14.3 | 5.0 ± 14.3 |  | -28.6 ± 9.2 | 15.6 ± 13.4 | 0.907 |
| HDL-C (mg/dL) | -0.8 ± 1.3 | -3.9 ± 1.8 |  | -1.3 ± 2.5 | 0.4 ± 1.9 | 0.235 |
| LDL-C (mg/dL) | -7.5 ± 2.7 | -5.2 ± 2.8 |  | -4.3 ± 4.0 | -8.0 ± 5.2 | 0.683 |
| ***MC4R rs476828*** | | | | | | |
|  | **TT genotype** | |  | **C carriers** | | **gene * diet**  **interaction**  ***p*-value** |
|  | **MeDiet**  **(n=17)** | **Control**  **(n=18)** |  | **MeDiet**  **(n=18)** | **Control**  **(n=18)** |  |
| Weight (kg) | -2.5 ± 0.4 | -0.3 ± 0.4 |  | -3.1 ± 0.5 | -0.5 ± 0.4 | 0.580 |
| BMI (kg/m^2^) | -1.1 ± 0.2 | -0.1 ± 0.2 |  | -1.3 ± 0.2 | -0.2 ± 0.2 | 0.682 |
| Skeletal muscle (kg) | -0.4 ± 0.2 | 0.0 ± 0.1 |  | -1.2 ± 0.5 | -0.5 ± 0.3 | 0.676 |
| Fat mass (kg) | -1.8 ± 0.5 | -0.4 ± 0.4 |  | -1.7 ± 0.6 | 0.4 ± 0.5 | 0.318 |
| Fat percentage (%) | -1.4 ± 0.7 | -0.4 ± 0.4 |  | -0.8 ± 0.8 | 0.8 ± 0.7 | 0.323 |
| WBC count (no./μL) | -0.67 ± 0.26 | -0.53 ± 0.28 |  | -0.36 ± 0.33 | 0.12 ± 0.24 | 0.575 |
| hsCRP (mg/L) | -0.4 ± 0.2 | 0.2 ± 0.5 |  | 2.1 ± 1.6 | 0.7 ± 0.7 | 0.199 |
| Fasting glucose (mg/dL) | 3.9 ± 1.9 | -0.3 ± 2.6 |  | -2.0 ± 2.3 | -0.1 ± 2.3 | 0.193 |
| Insulin (μIU/mL) | -2.8 ± 1.2 | -0.5 ± 1.2 |  | -1.5 ± 0.7 | -0.4 ± 1.3 | 0.653 |
| HOMA-IR | -0.60 ± 0.30 | -0.22 ± 0.35 |  | -0.40 ± 0.19 | -0.09 ± 0.51 | 0.923 |
| TC (mg/dL) | -9.1 ± 5.2 | -10.8 ± 5.5 |  | -8.5 ± 3.9 | -2.9 ± 3.9 | 0.262 |
| Triglycerides (mg/dL) | -46.4 ± 15.8 | 5.3 ± 14.5 |  | -17.9 ± 11.5 | 11.2 ± 16.0 | 0.530 |
| HDL-C (mg/dL) | 0.9 ± 1.7 | -2.9 ± 1.4 |  | -2.7 ± 1.6 | -2.3 ± 2.5 | 0.313 |
| LDL-C (mg/dL) | -7.3 ± 3.8 | -9.2 ± 3.6 |  | -5.6 ± 2.6 | -2.9 ± 3.3 | 0.310 |

Data are expressed as mean ± SEM. Gene-diet interaction *p*-values are calculated using a general linear regression model adjusting for age and initial BMI. Abbreviations: BMI, body mass index; hsCRP, high-sensitivity C-reactive protein; HDL-C, high-density lipoprotein cholesterol; HOMA-IR, homeostasis model of assessment-insulin resistance; LDL-C, low-density lipoprotein cholesterol; MeDiet, Mediterranean diet; TC, total cholesterol; WBC, white blood cell.
