# Supplementary material for: Effects of Single Nucleotide Polymorphisms and Mediterranean Diet in Overweight or Obese Postmenopausal Women With Breast Cancer Receiving Adjuvant Hormone Therapy: A Pilot Randomized Controlled Trial
Source: Front Nutr. 2022 Jul 1;9:882717. doi: 10.3389/fnut.2022.882717 (PMC9284001; doi:10.3389/fnut.2022.882717)
Supplement: Supplementary file 4 [file Table_4.docx]

**Table S4** Changes in body composition and metabolic parameters by dietary intervention.

|  | **MeDiet (n=35)** | **Control (n=36)** | ***p*-value** |
| --- | --- | --- | --- |
| Weight (kg) | -2.8 ± 0.3 | -0.4 ± 0.3 | <0.001 |
| BMI (kg/m^2^) | -1.2 ± 0.1 | -0.2 ± 0.1 | <0.001 |
| Skeletal muscle (kg) | -0.8 ± 0.3 | -0.2 ± 0.2 | 0.094 |
| Fat mass (kg) | -1.7 ± 0.4 | 0.0 ± 0.3 | <0.001 |
| Fat percentage (%) | -1.1 ± 0.5 | 0.2 ± 0.4 | 0.031 |
| WBC count (no./μL) | -0.51 ± 0.21 | -0.20 ± 0.19 | 0.303 |
| hsCRP (mg/L) | 0.9 ± 0.8 | 0.4 ± 0.4 | 0.557 |
| Fasting glucose (mg/dL) | 0.9 ± 1.6 | -0.2 ± 1.7 | 0.606 |
| Insulin (μIU/mL) | -2.2 ± 0.7 | -0.4 ± 0.9 | 0.131 |
| HOMA-IR | -0.50 ± 0.18 | -0.15 ± 0.31 | 0.337 |
| TC (mg/dL) | -8.8 ± 3.2 | -6.9 ± 3.4 | 0.551 |
| Triglyceride (mg/dL) | -31.7 ± 9.8 | 8.2 ± 10.7 | 0.007 |
| HDL –C (mg/dL) | -1.0 ± 1.2 | -2.6 ± 1.4 | 0.361 |
| LDL-C (mg/dL) | -6.4 ± 2.2 | -6.1 ± 2.5 | 0.764 |

Data are expressed as mean ± SEM. *p*-values are calculated by the analysis of covariance (ANCOVA), adjusted for age and initial BMI. Abbreviations: BMI, body mass index; CRP, C-reactive protein; HDL-C, high-density lipoprotein cholesterol; HOMA-IR, homeostasis model of assessment-insulin resistance; LDL-C, low-density lipoprotein cholesterol; MeDiet, Mediterranean diet; TC, total cholesterol; WBC, white blood cell.
