# Supplementary material for: Effects of Single Nucleotide Polymorphisms and Mediterranean Diet in Overweight or Obese Postmenopausal Women With Breast Cancer Receiving Adjuvant Hormone Therapy: A Pilot Randomized Controlled Trial
Source: Front Nutr. 2022 Jul 1;9:882717. doi: 10.3389/fnut.2022.882717 (PMC9284001; doi:10.3389/fnut.2022.882717)
Supplement: Supplementary file 5 [file Table_5.docx]

**Table S5** Changes in nutrient intake and physical activity by dietary intervention.

|  | **MeDiet (n=35)** | | |  | **Control (n=36)** | | |
| --- | --- | --- | --- | --- | --- | --- | --- |
|  | **Baseline** | **8 weeks** | ***p*-value** |  | **Baseline** | **8 weeks** | ***p*-value** |
| Calorie (kcal/day) | 1289.2 ± 65.6 | 1221.1 ± 44.8 | 0.369 |  | 1448.6 ± 66.4 | 1315.9 ± 61.1 | 0.082 |
| Carbohydrate (%) | 62.5 ± 1.9 | 53.8 ± 1.0 | <0.001 |  | 59.5 ± 1.6 | 57.8 ± 1.3 | 0.328 |
| Protein (%) | 15.8 ± 0.8 | 18.7 ± 0.5 | 0.003 |  | 16.5 ± 0.7 | 15.8 ± 0.5 | 0.325 |
| Total fat (%) | 22.2 ± 1.4 | 29.2 ± 0.7 | <0.001 |  | 23.6 ± 1.4 | 26.4 ± 1.2 | 0.079 |
| SFA (%) | 5.3 ± 0.5 | 5.6 ± 0.5 | 0.660 |  | 5.5 ± 0.5 | 6.3 ± 0.6 | 0.304 |
| MUFA (%) | 5.7 ± 0.5 | 14.0 ± 0.6 | <0.001 |  | 5.3 ± 0.5 | 6.3 ± 0.6 | 0.195 |
| PUFA (%) | 4.6 ± 0.4 | 6.9 ± 0.5 | 0.001 |  | 4.6 ± 0.4 | 5.1 ± 0.4 | 0.368 |
| Trans fat (%) | 0.22 ± 0.03 | 0.12 ± 0.03 | 0.010 |  | 0.24 ± 0.03 | 0.33 ± 0.04 | 0.107 |
| K-MEDAS score | 6.2 ± 0.3 | 12.3 ± 0.2 | <0.001 |  | 5.9 ± 0.3 | 6.4 ± 0.3 | 0.051 |
| GLTEQ score | 27.6 ± 4.8 | 25.9 ± 3.6 | 0.723 |  | 16.2 ± 2.7 | 23.7 ± 3.8 | 0.047 |

Data are expressed as mean ± SEM. *p*-values are calculated by the paired t-test. Abbreviations: GLTEQ, Godin Leisure-Time Exercise Questionnaire; K-MEDAS, Korean version of the Mediterranean Diet Adherence Screener; MeDiet, Mediterranean diet; MUFA, monounsaturated fatty acid; PUFA, polyunsaturated fatty acid; SFA, saturated fatty acid.
